# A Novel Dopamine Transporter Inhibitor CE-123 Improves Cognitive Flexibility and Maintains Impulsivity in Healthy Male Rats

**DOI:** 10.3389/fnbeh.2017.00222

**Published:** 2017-11-27

**Authors:** Agnieszka Nikiforuk, Predrag Kalaba, Marija Ilic, Volker Korz, Vladimir Dragačević, Judith Wackerlig, Thierry Langer, Harald Höger, Joanna Golebiowska, Piotr Popik, Gert Lubec

**Affiliations:** ^1^Department of Behavioral Neuroscience and Drug Development, Institute of Pharmacology, Polish Academy of Sciences, Kraków, Poland; ^2^Department of Pharmaceutical Chemistry, Faculty of Life Sciences, University of Vienna, Vienna, Austria; ^3^Center for Brain Research, Medical University of Vienna, Vienna, Austria; ^4^Core Unit of Biomedical Research, Division of Laboratory Animal Science and Genetics, Medical University of Vienna, Vienna, Austria; ^5^Paracelsus Medical University, Salzburg, Austria

**Keywords:** cognitive flexibility, novel DAT inhibitors, cognitive enhancement, diminished side effects, impulsivity

## Abstract

Reduced cognitive abilities are often characterized by an impairment of flexibility, i.e., the ability to switch from learned rules or categories that were important in certain contexts to different new modalities that rule the task. Drugs targeting the dopamine transporter (DAT) are widely used for their potential to enhance cognitive abilities. However, commercially available drugs are of limited specificity for DAT, blocking also noradrenaline and serotonine transporters, that can lead to unwanted side effects in healthy subjects. Therefore, we tested a newly synthetized compound (CE-123) with higher specificity for DAT in male rats in an attentional set-shifting task (ASST), that proves for cognitive flexibility and a 5-choice serial-reaction time task (5-CSRTT) assessing visuospatial attention and impulsivity. Treated rats at a dose of 0.3 and 1.0 but not 0.1 mg/kg bodyweight showed reduced extra-dimensional shifts in the ASST compared to controls indicating increased cognitive flexibility. Rats treated with R-Modafinil, a commercially available DAT inhibitor at a dose of 10 mg/kg bodyweight showed increased premature responses, an indicator of increased impulsivity, during a 10 s but not a 2.5, 5, or 7.5 s intertrial interval when compared to vehicle-treated rats in the 5-CSRTT. This was not found in rats treated with CE-123 at the same dose as for R-Modafinil. Visuospatial attention, except premature responses, did not differ between R-Modafinil and CE-123-treated rats and their respective controls. Thus, CE-123 increased cognitive flexibility with diminished impulsivity.

## Introduction

Reduced cognitive flexibility is a major symptom in the decline of cognitive ability induced by aging or mental diseases. Cognitive flexibility is, besides other neurotransmitters, also regulated by dopamine and especially through dopamine receptor type 1 (D1R) activity, as assessed by the attentional set-shifting task (ASST), a widely used test for cognitive flexibility (Gauthier et al., 2014; Nikiforuk et al., 2016). There is, however, also evidence that multiple dopamine receptor subtypes regulate set-shifting (Floresco et al., 2006). DAT inhibition blocks the reuptake of dopamine from extracellular space into the synapse, thus increasing the amount of available dopamine which enhances the probability of dopamine receptor activation. However, the specificity for DAT of most of the DAT inhibitors is limited as they significantly bind also to noradrenaline and serotonine transporters and these substances can cause adverse side effects in healthy subjects (Kumar, 2008; Dolder et al., 2017), including increased impulsivity (Waters et al., 2005), although there is evidence that other neurotransmitters such as noradrenaline regulate set-shifting (Tait et al., 2007). Therefore, we synthetized a novel compound CE-123, targeting the DAT with high specificity, and tested the effects on cognitive flexibility by using an ASST. Visuospatial attention and impulsivity were tested by employing a 5-choice serial reaction time task (5-CSRTT) in healthy young male rats. Since the improving effects of Modafinil upon attentional set shifting is already known (Goetghebeur and Dias, 2009) we focused here on the novel compound.

Similar to spatial working memory cognitive flexibility implicates, besides other brain regions, also involves prefrontal processes (Tait et al., 2014), however, by different optimal levels of dopamine. An “inverted U-shaped” relation between prefrontal dopamine and spatial working memory with poor performance at very low and very high dopamine concentrations has been found in rodents and humans (Zahrt et al., 1997; Cools and D’Esposito, 2011), whereas cognitive flexibility is regulated in a linear dose-dependent pattern (Fallon et al., 2015). Drugs targeting the dopamine transporter (DAT) are widely used to enhance cognitive abilities in aged or diseased subjects (Bobo et al., 2011; Battleday and Brem, 2015; Dolder et al., 2017). The most commonly used DAT targeting drug Modafinil, a wake promoting agent, has also been widely tested as a cognitive enhancer (Turner et al., 2003) and in the therapy of schizophrenia-related cognitive impairments (Turner et al., 2004a; Bobo et al., 2011) as well as in attention deficit hyperactivity disorder (Turner et al., 2004b; Wang et al., 2017). However, the effects in healthy subjects are less well studied and the results are controversial (Marchant et al., 2009; Battleday and Brem, 2015; Ikeda et al., 2017).

## Materials and Methods

### Synthesis of Racemic CE-123

#### Synthesis of [(Diphenylmethyl)sulfanyl] methanimideamide Hydrobromide

Diphenylmethanol (30.92 g, 168 mmol) and thiourea (14.78 g, 194 mmol) were added into a 1 l two-neck round-bottom flask and dissolved in 150 ml of methanol. The reaction mixture was refluxed for 0.5 h. Then, HBr (48%, 87.4 ml, 772 mmol) was added drop-wise over 1 h and the reaction mixture was stirred under reflux for additional 2.5 h. After cooling to room temperature, methanol was removed under reduced pressure. The pale yellow product was suspended in 100 ml of dichloromethane and stirred for 0.5 h at room temperature. Upon filtration under reduced pressure the residue was washed with 140 ml of water and stirred for additional 0.5 h at room temperature. The product was filtered under reduced pressure and dried under high-vacuum to yield 38.4 g of a white, powdered solid (yield 71%).

#### Synthesis of 5-(Chloromethyl)thiazole Hydrochloride

In a 500 ml round bottomed flask 10 g (86.8 mmol) of 5-hydroxymethylthiazol was dissolved in 75 ml of dichloromethane and the mixture was cooled to 0°C. Then, 6.3 ml of thionyl chloride (1 equivalent, 86.8 mmol) was added drop-wise and the reaction mixture was stirred at room temperature overnight. Then the product was concentrated under reduced pressure and dried under high vacuum to yield 14.6 g of an orange solid (yield > 95%).

#### Synthesis of 5-((Benzhydrylthio)methyl)thiazole

[(Diphenylmethyl)sulfanyl]methanimideamide hydrobromide 32.47 g (86.4 mmol) was dissolved in 150 ml of methanol. Afterward, 14.6 g (86.4 mmol) of 5-(chloromethyl)thiazole hydrochloride and 59.6 g (5 equivalents, 430 mmol) of potassium carbonate were added to the reaction mixture and stirred for 2 days at room temperature. Subsequently, methanol was evaporated and 250 ml of water was added to the residual mixture and extracted with ethyl acetate, dried over Na_2_SO_4_, and filtered off. The organic solution was concentrated under reduced pressure to achieve 21.1 g of a solid product (yield 82.3%).

#### Synthesis of 5-((Benzhydrylsulfinyl)methyl)thiazole (CE-123)

5-((Benzhydrylthio)methyl)thiazole (21.09 g, 71 mmol) was dissolved in 50 ml of glacial acetic acid. Then, 8.19 ml (71 mmol) of 30% H_2_O_2_ was dropped into the solution and stirred for 12 h. The acid solution was neutralized with 5% sodium bicarbonate in an ice-bath. Reaction products were extracted (3×) from the neutral solution with 150 ml of ethyl acetate. Organic extracts were collected, combined, dried over Na_2_SO_4_, and filtered off. Then, the solvent was separated under reduced pressure to obtain a brownish solid which was suspended in 400 ml of diethyl ether and stirred under reflux for 2 h. The etheric solution was slowly cooled +4°C and the resulting product was filtered under reduced pressure and washed (3×) with 50 ml of cold diethyl ether and dried under high vacuum overnight to obtain 13.178 g of a white powdered product (yield 59.2%).

### Analytical Methods

NMR spectra were recorded on a Bruker Avance 500 NMR Spectrometer (UltraShield) using a 5 mm switchable probe (PA BBO 500SB BBF-H-D-05-Z, 1H, BB = 19F and 31P – 15N) with *z*-axis gradients and automatic tuning and matching accessory (Bruker BioSpin). The resonance frequency for ^1^H NMR was 500.13 MHz and for ^13^C NMR 125.75 MHz. All measurements were performed for a solution in fully deuterated chloroform or methanol at 298 K. Standard 1D and gradient-enhanced (ge) 2D experiments, like double quantum filtered (DQF) COSY, NOESY, HSQC, and HMBC, were used as supplied by the manufacturer. Chemical shifts are referenced internally to the residual, non-deuterated solvent signal for chloroform ^1^H (δ 7.26 ppm) or methanol ^1^H (δ 3.31 ppm) and to the carbon signal of the solvent for chloroform ^13^C (δ 77.00 ppm) or methanol ^13^C (δ 49.00 ppm).

HRESIMS spectra were obtained on a maXis HD ESI-Qq-TOF mass spectrometer (Bruker Daltonics, Bremen, Germany). Samples were dissolved to 20 μg/ml in MeOH and directly infused into the ESI source at a flow rate of 3 μl/min with a syringe pump. The ESI ion source was operated as follows: capillary voltage: 0.9–4.0 kV (individually optimized), nebulizer: 0.4 bar (N_2_), dry gas flow: 4 l/min (N_2_), and dry temperature: 200°C. Mass spectra were recorded in the range of *m/z* 50–1550 in the positive-ion mode. The sum formulas were determined using Bruker Compass DataAnalysis 4.2 based on the mass accuracy (Δ*m/z* ≤ 2 ppm) and isotopic pattern matching (SmartFormula algorithm).

The purity of the compounds was determined by HPLC on an UltiMate 3000 series system equipped with VWD detector (Dionex/Thermo Fisher Scientific, Germering, Germany) and on a LC-2010A HT Liquid Chromatograph device (Shimadzu Corporation, Tokyo, Japan). Separation was carried out on an Acclaim 120 C18, 2.1 × 150 mm, 3 μm HPLC column (Thermo Fisher Scientific) using LC-MS-grade water and acetonitrile as mobile phase A and B, respectively. The sample components were separated and eluted with a linear gradient from 10 to 90% B in 25 min followed by an isocratic column cleaning and re-equilibration step. The flow rate was 0.2 ml/min and the column oven temperature was set to 25°C. The purity was determined by HPLC with UV detector (254 nm), as being the ratio of the peak area of the compound and the total peak areas (i.e., the sum of the areas of all peaks that were not present in the solvent blank).

### Separation of Racemic CE-123 into Individual Enantiomers

To separate racemic CE-123 to individual enantiomers, S and R, we first performed screening on an HPLC device using chiral analytical Chiralpak IA, Chiralpak IC, Chiralpak ADH; (Daicel Inc., Tokyo, Japan) columns to look for suitable conditions. Chiralpak IA column and 100% EtOH and 100% ACN provided appropriate results (Supplementary Figure 1).

### Semi-preparative Separation of CE-123 Enantiomers

Racemic CE-123 was dissolved in 100% acetonitrile to a concentration of 12 mg/ml. Stacked injections were performed every sixth minutes on a Shimadzu 10AVP HPLC System (Shimadzu Corporation, Tokyo, Japan) equipped with Chiralpak IA semi-preparative column (10 mm diameter × 20 mm length). Absolute ethanol was used as a mobile phase and eight injections can be made per each 60 min of chromatographic run (Supplementary Figure 2). The chromatographic runs were repeated until the amount of enantiomeric S-CE123 required for animal experiments was obtained.

Collected fractions of individual S-enantiomer were pooled, concentrated under reduced pressure, and dried under high vacuum.

### Reuptake Inhibition Assay

Dulbecco’s modified Eagle’s medium (DMEM), trypsin, and fetal calf serum (FCS) were purchased from Sigma–Aldrich Handels GmbH (Austria). [^3^H]5-HT (Hydroxytryptamine creatinine sulfate; 5-[1,2-^3^H[N]]; 27.8 Ci/mmol), [^3^H]DA (Dihydroxyphenylethylamine; 3,4-[ring-2,5,6-^3^[H]]-Dopamine; 36.6 Ci/mmol), and [^3^H]MPP^+^ (Methyl-4-phenylpyridinium iodide; 1-[methyl-3H]; 80 Ci/mmol) were purchased from Perkin Elmer, Boston, MA, United States.

HEK293 cells stably expressing human isoforms of the DAT, norepinephrine transporter (NET), and serotonin transporter (SERT) were used for reuptake inhibition assays. Effect of CE-123 enantiomers on substrate reuptake was analyzed as described by Sucic et al. (2010). In brief, cells were seeded on 96-well plates precoated with poly-D-lysine (PDL) (5 × 10^4^ cells/well) 24 h prior to the experiment. Each well was washed with 100 μl of Krebs-HEPES buffer (KHB; 10 mM HEPES, 120 mM NaCl, 3 mM KCl, 2 mM CaCl_2_⋅2H_2_O, 2 mM MgCl_2_⋅6H_2_O, 5 mM D-(+)-glucose monohydrate, pH 7.3). Cells were preincubated 5 min in KHB containing increasing concentrations of CE-123 enantiomers. CE-123 enantiomers were dissolved first in 99.9% dimethyl sulfoxide (DMSO) and subsequently diluted in KHB. Afterward, cells were incubated in KHB containing increasing concentrations of compound with addition of 0.2 μM [^3^H]-dopamine (for HEK-DAT), 0.05 μM [^3^H]MPP^+^ (for HEK-NET), and 0.2 μM [^3^H]5-HT (for HEK-SERT). Incubation times were 1 min for HEK-DAT and HEK-SERT and 3 min for HEK-NET. For determination of unspecific uptake in HEK-DAT and HEK-NET 10 μM mazindol was used and 10 μM paroxetine was used for HEK-SERT. After incubation at room temperature, reaction was stopped by the addition of 100 μl of ice-cold KHB. Finally, cells were lysed with 300 μl of 1% SDS and released radioactivity was measured by a liquid scintillation counter (Tri-carb-2300TR, Perkin Elmer, Boston, MA, United States).

### Animals

Male Sprague-Dawley rats (Charles River, Sulzfeld, Germany) weighing 350–400 g on arrival were housed in a temperature-controlled (21 ± 1°C) and humidity-controlled (40–50%) colony room under a 12/12 h light/dark cycle (lights on at 06:00 h). The rats were group-housed (4–5 rats/cage) with a mild food restriction (17 g of food pellets per day) for at least 1 week prior to testing and with free access to water. Behavioral testing was performed during the light phase of the light/dark cycle.

The experiments were conducted in accordance with the NIH Guide for the Care and Use of Laboratory Animals and approved by the II Local Ethics Committee for Animal Experiments at the Institute of Pharmacology, Polish Academy of Science, Krakow, Poland.

### Attentional Set-Shifting Task (ASST)

#### Apparatus

Testing was conducted in a Plexiglas apparatus (length × width × height: 38 × 38 × 17 cm) with the grid floor and wall dividing half of the length of the cage into two sections. During testing, one ceramic digging pot (internal diameter of 10.5 cm and a depth of 4 cm) was placed in each section. Each pot was defined by a pair of cues along with two stimulus dimensions. To mark each pot with a distinct odor, 5 μl of a flavoring essence (Dr. Oetker^®^, Poland or The Body Shop, United Kingdom) was applied to a piece of blotting paper fixed to the external rim of the pot immediately prior to use. A different pot was used for each combination of digging medium and odor; only one odor was ever applied to a given pot. The bait (one-half of a Honey Nut Cheerio, Nestle^®^) was placed at the bottom of the “positive” pot and buried in the digging medium. A small amount of powdered Cheerio was added to the digging media to prevent the rat from trying to detect the buried reward by its smell.

### ASST Procedure

As described previously (Nikiforuk et al., 2015), the procedure lasted 3 days for each rat.

#### Day 1, Habituation

Rats were habituated to the testing area and trained to dig in the pots filled with sawdust to retrieve the food reward. Rats were transported from the housing facility to the testing room where they were presented with one unscented pot (filled with several pieces of Cheerios) in their home cages. After the rats had eaten the Cheerio from the home cage pot, they were placed in the apparatus and given three trials to retrieve the reward from both of the sawdust-filled baited pots. With each exposure, the bait was covered with an increasing amount of sawdust.

#### Day 2, Training

Rats were trained on a series of simple discriminations (SD) to a criterion of six consecutive correct trials. For these trials, rats had to learn to associate the food reward with an odor cue (e.g., arrack vs. orange, both pots filled with sawdust) and/or a digging medium (e.g., plastic balls vs. pebbles, no odor). All rats were trained using the same pairs of stimuli. The positive and negative cues for each rat were presented randomly and equally. These training stimuli were not used again in later testing trials.

#### Day 3, Testing

Rats performed a series of discriminations in a single test session. The first four trials at the beginning of each discrimination phase served as discovery period: the rats were allowed to dig in both of the pots. In subsequent trials, if the rat started to dig in the incorrect pot, an error was recorded, and the trial was terminated. Digging was defined as any distinct displacement of the digging media with either the paw or the nose; the rat could investigate a digging pot by sniffing or touching without displacing material. Testing was continued at each phase until the rat reached the criterion of six consecutive correct trials, after which testing proceeded to the next phase.

In the SD involving only one stimulus dimension, the pots differed along one of two dimensions (e.g., digging medium). For the compound discrimination (CD), the second (irrelevant) dimension (i.e., odor) was introduced but the correct and incorrect exemplars of the relevant dimension remained constant. For the reversal of this discrimination (Rev 1), the exemplars and relevant dimension were unchanged, but the previously correct exemplar was now incorrect and vice versa. The intra-dimensional (ID) shift was then presented, comprising new exemplars of both, the relevant and irrelevant dimensions with the relevant dimension remaining the same as previously. The ID discrimination was then reversed (Rev 2) so that the formerly positive exemplar became the negative one. For the extra-dimensional (ED) shift, a new pair of exemplars was again introduced, but this time a relevant dimension was also changed. Finally, the last phase was the reversal (Rev 3) of the ED discrimination. The exemplars were always presented in pairs and varied so that only one animal within each treatment group received the same combination. The assignment of each exemplar in a pair as being positive or negative at a given phase and the left-right positioning of the pots in the test apparatus on each trial were randomized. The following pairs of exemplars were used: Pair 1: odor: spicy vs. vanilla, medium: cotton wool vs. crumpled tissue; Pair 2: odor: lemon vs. almond, medium: shredded pipette tips vs. wooden sticks; and Pair 3: odor: rum vs. cream, medium: shredded papers vs. silk. The assignment of each exemplar in a pair as being positive or negative at a given phase and the left-right positioning of the pots in the test apparatus on each trial were randomized. The total number of rats that were subjected to the SD training was 45. The rats that had completed initial training (*N* = 37) were drug- (or vehicle) treated and tested in the ASST.

### Data Collection

The number of trials required to achieve the criterion of six consecutive correct responses was recorded for each rat and for each discrimination phase of the ASST.

### Five-Choice Serial Reaction Time Task (5-CSRTT)

#### Apparatus

Eight-five choice operant chambers (Med Associates, United States), measuring 56 × 56 × 40.5 cm, were housed in sound-attenuated and ventilated cubicles. In each chamber, an array of five square nose-poke holes (2.5 × 2.5 × 2.5 cm) was arranged on a curved panel and raised 2.5 cm from the grid floor. Each hole was equipped with an infrared detector and a yellow stimulus light at its rear. The food magazine, equipped with photocell beams and light, was located on the opposite wall. Food pellets (45 mg, Bioserves, United States) were delivered via a dispenser connected to the food magazine. A house light was located 17 cm above the top edge of the food magazine. Online control of the apparatus and data collection were performed using MED-PC (Med Associates, United States).

#### 5-CSRTT Procedure

##### Magazine training

In the initial training phase, rats had to learn that food pellets were available in the magazine (Nikiforuk et al., 2015). On the first day, rats were habituated to the operant chambers for 15 min. During this habituation session, the food magazine was filled with several pellets. Next, rats were given magazine training sessions, in which every head entry into a food magazine resulted in a pellet delivery. Once all of the rats ate 100 food pellets within a session (which usually took one or two sessions), the training proceeded to the next stage.

##### Pre-training

In this phase, rats had to learn to associate a nose-poke response into an illuminated hole with a pellet delivery. All of the five holes were illuminated, and a response in any aperture was rewarded by a food pellet. This procedure was continued daily until rats obtained 100 pellets within a session.

Each session began with the illumination of the house light and delivery of a food pellet. A nose-poke into the magazine tray initiated the first trial, which consisted of an inter-trial interval (ITI) followed by the random illumination of one of the five holes for a fixed interval (stimulus duration, Sd). If a nose-poke was registered in the illuminated hole before the end of the limited hold (Lho, i.e., a fixed interval after Sd), a pellet was delivered and a correct trial was registered. An incorrect response or a failure to respond within the required period (omission) resulted in a time out (TO) period, in which the house light was extinguished. Responding to any of the five holes during the ITI (premature response) also resulted in a TO. For the first session of training, the stimulus duration and limited hold periods were both set at 60 s, and the ITI and TO were at 2 s each. These variables were gradually altered during training, and the final test parameters were as follows: Sd = 1 s, Lho = 5 s, ITI = 5 s, and TO = 5 s. Rats were trained until they reached the following criteria: accuracy > 70%, omissions < 30%, and stable baseline performance across five consecutive sessions (that took approximately 60 training sessions). Each session lasted until 100 trials had been completed or a duration of 60 min. During the testing session, animals were exposed to variable ITI (vITI) durations (2.5, 5, 7.5, and 10 s). Equal numbers (i.e., 25) of each of the four ITIs were randomly presented during the 100 trial session over a 60-min period. During testing sessions, all rats completed 100 trials within 60 min. The total number of rats that were subjected to the training was 50. The rats that had completed initial training (*N* = 49) were assigned randomly to drug and dose. Each animal received two injections with a 1-week drug-free period between testing sessions. No animal received the same treatment twice.

### Data Collection

The following parameters were recorded in each session: percent accuracy (number of correct responses divided by the sum of correct and incorrect responses × 100), number of omissions (total number of trials omitted during a 100-trial session), premature responses (total number of responses performed during the ITIs), perseverant responses (total number of responses emitted after a correct response had been made), correct response latency (time from the stimulus onset to a correct response), reward latency (time from a correct response to the retrieval of food from the magazine), and head entries (total number of head entries to the magazine).

### Drug Administration

CE-123 was dissolved in an aqueous 10% Cremophor solution and R-Modafinil was dissolved in distilled water. Drugs or vehicle (physiological saline or 10% Cremophor solution) were administered intraperitoneally (IP) in a volume of 1 ml/kg of body weight. Drugs were administered 30 min prior testing.

### Statistics

#### ASST

Data were analyzed using a two-way mixed-design ANOVA with the treatment as a between-subject factor and the discrimination phase as a repeated measure. 5-CSRTT: Data were subjected to two-way mixed-design ANOVAs with the respective drug treatment as a between-subject factor and vITIs as a repeated measure.

*Post hoc* comparisons were performed using the Newman–Keuls test. The statistical analyses were performed using Statistica 12.0 for Windows. Statistical significance was set at *p* < 0.05.

## Results

### Characterization of Racemic CE-123 Preparation and CE-123 Enantiomers

**FIGURE 1:**
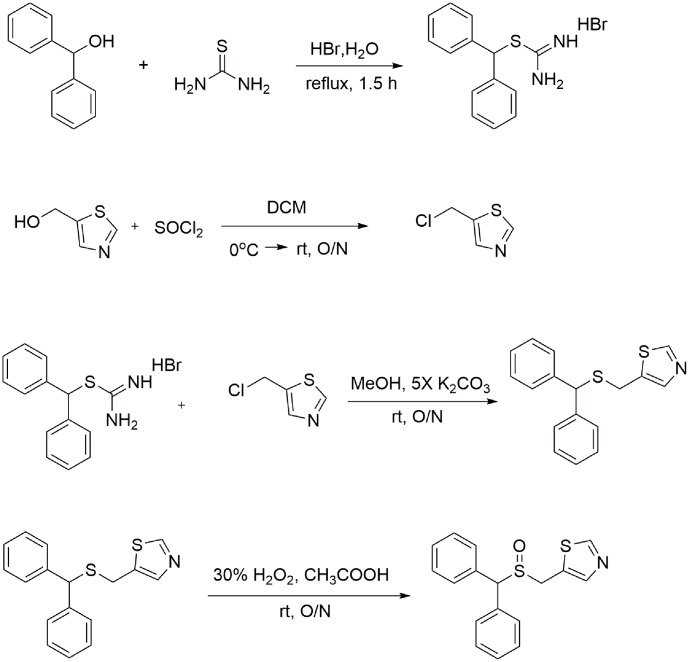
Synthesis of the CE-123 race mate. In the first step diphenylmethanol is reacted with thiourea to yield thiouronium salt. In the second step thiazol-5yl-methanol is converted to 5-(chloromethyl) thiazole. The third step is the alkylation reaction of thiouronium salt with 5-(chloromethyl) thiazole to yield 5-((benzhydrylthio) methyl) thiazole (un-oxidized CE-123 precursor). Finally, 5-((benzhydrylthio)methyl) thiazole is oxidized with 30% H_2_O_2_ in gl. acetic acid to yield the final product 5-((benzhydrylsulfinyl)methyl) thiazole (CE-123).

Synthesis steps are given in **Figure 1**. The overall purity of the racemic compound, determined by a C18 analytical column-based HPLC method using reversed-phase chromatography conditions was 98.3% (Supplementary Figure 3). The experimental molecular mass of the CE-123 was determined from the precursor ion HRESIMS *m/z* 314.0667 [M+H]^+^ (calculated for C_17_H_16_NOS_2_^+^, 314.0668, Δ = 0.3 ppm) (Supplementary Figure 4).

Proton and carbon NMR of the race mate results were

^1^H NMR (500 MHz, CDCl_3_-d, 23°C): δ = 8.86 (s, 1H, C*H*, thiazole-2), 7.67 (s, 1H, C*H*, thiazol-4), 7.39 (2× C*H*-2,6, phenyls), 7.45 (C*H*-3,5, phenyl), 7.39 (C*H*-3,5, phenyl), 7.36 (2× C*H*-4, phenyls), 4.65 (s, 1H, C*H*), δ = 4.13, 3.89 (AB, 2H, C*H*_2_) (Supplementary Figure 5). ^13^C{^1^H}NMR (125.75 MHz, CDCl_3_-d, 23°C): δ = 46.64 (*C*H_2_), 69.95 (*C*H), 128.54 (*C*-4, phenyl), 128.77 (*C*-4, phenyl), 129.33 (2× *C*-3,5, phenyl), 129.52 (2×*C*-3,5, phenyls), 128.66 (*C*-2,6, phenyl), 128.81 (*C*-2,6, phenyl), 134.05 (*C*_q_-1, phenyl), 134.74 (*C*_q_-1, phenyl), 124.89 (*C*-2, thiazole), 144.18 (*C*-4, thiazole), 154.97 (*C*-5, thiazole) (Supplementary Figure 6).

The overall purity of the enantiomers, determined by a C18 analytical column-based HPLC method using reversed-phase conditions was 99.37% for less retained enantiomer S (Supplementary Figure 7) and 99.24% for more retained enantiomer R (Supplementary Figure 8).

Each enantiomer was analyzed for enantiomeric purity on a chiral column and for the enantiomer S, used in the current study, the purity was 97.97% (Supplementary Figure 9), while for the enantiomer R, purity was 99.15% (Supplementary Figure 10).

### NMR Results of the Enantiomers

#### Enantiomer S

^1^H (500 MHz, CDCl_3_) δ 8.84 (s,1H), 7.67 (s,1H), 7.47–7.31 (m, 10H), 4.66 (s,1H), 4.11 (d, *J* = 15 Hz, 1H), 3.87 (d, *J* = 15 Hz, 1H) (Supplementary Figure 11); ^13^C (125.75 MHz, CDCl_3_) δ 154.9 (CH), 144.2 (CH), 134.8 (C), 134.1 (C), 129.5 (2CH), 129.3 (2CH), 128.8 (2CH), 128.7 (CH), 128.6 (2CH), 128.5 (CH), 125.1 (C), 70.1 (CH), 46.8 (CH_2_) (Supplementary Figure 12). Enantiomer R: ^1^H (500 MHz, CDCl_3_) δ 8.85 (s,1H), 7.67 (s,1H), 7.47–7.31 (m, 10H), 4.66 (s,1H), 4.11 (d, *J* = 15 Hz, 1H), 3.87 (d, *J* = 15 Hz, 1H) (Supplementary Figure 13); ^13^C (125.75 MHz, CDCl_3_) δ 154.9 (CH), 144.2 (CH), 134.8 (C), 134.1 (C), 129.5 (2CH), 129.3 (2CH), 128.8 (2CH), 128.7 (CH), 128.6 (2CH), 128.5 (CH), 125.1 (C), 70.1 (CH), 46.8 (CH_2_) (Supplementary Figure 14).

Mass spectrometry providing high-resolution mass spectra confirmed the molecular mass of both enantiomers, as being HRESIMS m/z 314.0669 [M+H]^+^ (calculated for C_17_H_16_NOS_2_^+^, 314.0668, Δ = -0.2 ppm) for enantiomer S (Supplementary Figure 15) and HRESIMS m/z 314.0669 [M+H]^+^ (calculated for C_17_H_16_NOS_2_^+^, 314.0668, Δ = -0.1 ppm) for enantiomer R (Supplementary Figure 16).

Pharmacokinetic characteristics for R-Modafinil and CE-123 enantiomer S are given in Supplementary Table 1. Reuptake inhibition assay results as a measure for DAT specificity are given in Supplementary Figure 17.

### ASST

**FIGURE 2:**
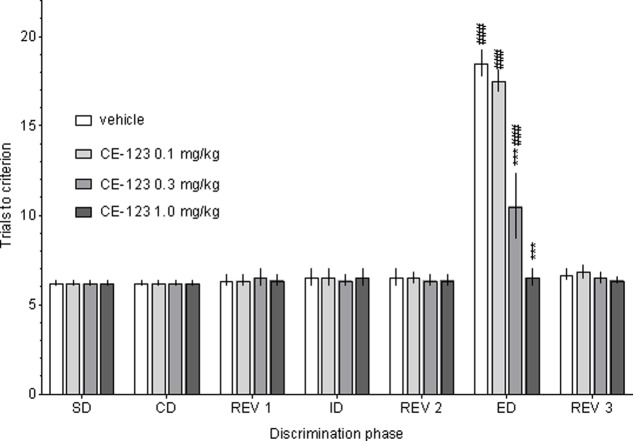
The effects of CE-123 on rat performance in the Attentional Set-Shifting Task (ASST). CE-123 (0, 0.1, 0.3, or 1 mg/kg) was administered to Sprague-Dawley rats IP 30 min before the task. The results represent the means ± SEM for the number of trials required to reach the criterion of six consecutive correct trials for each of the discrimination phases. *N* = 6 rats per group. *Symbols*: ^∗∗∗^*p* < 0.001 vs. ED performance in the vehicle-treated group. ^###^*p* < 0.001 vs. the given groups’ ID performance. SD, simply discrimination; CD, compound discrimination; Rev 1, reversal 1; ID, intra-dimensional shift; Rev 2, reversal 2; ED, extra-dimensional shift; Rev 3, reversal 3.

**FIGURE 3:**
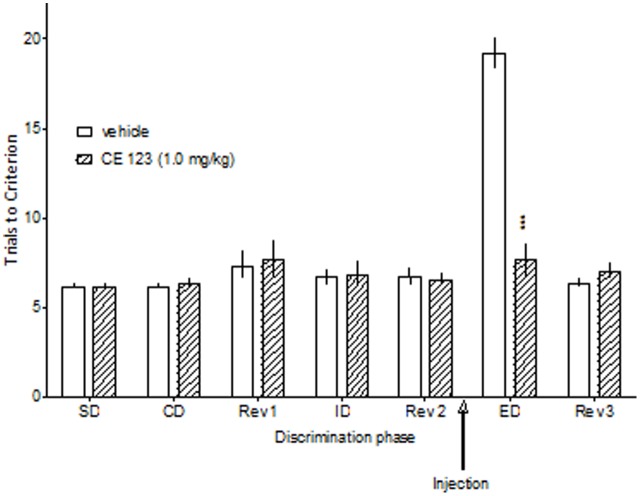
The effect of CE-123 administered after attentional set acquisition. CE-123 (0 or 1 mg/kg) was administered to Sprague-Dawley rats IP 30 min before the ED stage. The results represent the means ± SEM for the number of trials required to reach the criterion of six consecutive correct trials for each of the discrimination phases. *N* = 6 rats per group. Symbols: ^∗∗∗^*p* < 0.001 vs. ED performance in the vehicle-treated group.

Control rats required significantly more trials to reach the criterion on the ED than the ID stage of the task, demonstrating that they had formed an attentional set toward the relevant dimension before ED discrimination stage (**Figure 2**). There was a significant two-way ANOVA interaction between the discrimination phase and CE-123 treatment: *F*(18,120) = 20.01, *p* < 0.001. *Post hoc* analysis revealed that the acute administration of CE-123 (0.3 and 1.0 mg/kg) significantly and specifically reduced the number of trials to criterion during the ED phase. There was no significant drug effect during any other discrimination stage (**Figure 3**). CE-123 (1.0 mg/kg) also facilitated ED set shifting when administered after the acquisition of the attentional set (i.e., immediately after the Rev 2 stage), which indicates that the ED improvement is not due to impairment in attentional set formation (**Figure 3**). A two-way ANOVA indicated a significant interaction between the discrimination phase and CE-123: *F*(6,60) = 25.93, *p* < 0.001.

### 5-CSRTT

**FIGURE 4:**
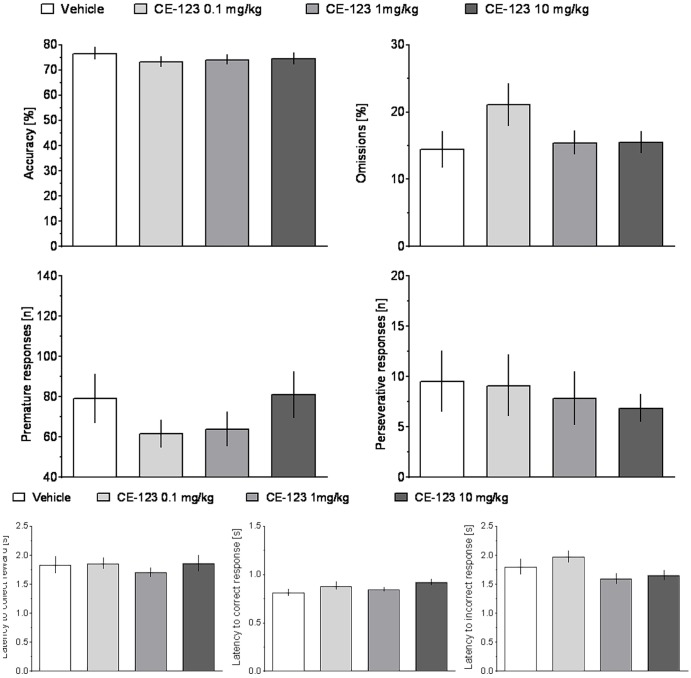
The effects of CE-123 on rat performance in the 5-choice serial reaction time task (5-CSRTT). CE-123 (0, 0.1, 1, or 10 mg/kg) was administered to rats IP 30 min before the task. *N* = 12–13 rats per group. Sprague-Dawley rats were exposed to vITI durations (2.5, 5, 7.5, and 10 s), equal numbers (i.e., 25) of each of the four ITIs were randomly presented during the 100 trial session over a 60-min period. The results represent the means ± SEM for total session without distinction on the individual ITIs.

ANOVA did not reveal significant effects of treatment or treatment × ITI interaction on any measure in rats treated with CE-123 as compared to vehicle controls (**Figure 4** and **Table 1**).

**Table 1:** Overview of the statistical results for different parameters of the 5-CSRTT experiments of rats treated with CE-123 or R-Modafinil.

ANOVA	ITI	Treatment	ITI × Treatment
**CE-123**			
Accuracy	*F*(3,135) = 14.21, *p* < 0.001	*F*(3,45) = 0.49, NS	*F*(9,135) = 1.39, NS
Omissions	*F*(3,135) = 56.66, *p* < 0.001	*F*(3,45) = 1.64, NS	*F*(9,135) = 0.96, NS
Premature	*F*(3,135) = 131.07, *p* < 0.001	*F*(3,45) = 0.24, NS	*F*(9,135) = 1.08, NS
Perseverant	*F*(3,135) = 0.89, NS	*F*(3,45) = 0.20, NS	*F*(9,135) = 1.14, NS
Latency reward	*F*(3,135) = 17.18, *p* < 0.001	*F*(3,45) = 0.62, NS	*F*(9,135) = 1.55, NS
Latency correct	*F*(3,135) = 8.03, *p* < 0.001	*F*(3,45) = 2.12, NS	*F*(9,135) = 1.44, NS
Latency incorrect	*F*(3,135) = 0.26, NS	*F*(3,45) = 0.78, NS	*F*(9,135) = 0.58, NS
**R-Modafinil**			
Accuracy	*F*(3,135) = 10.82, *p* < 0.001	*F*(3,45) = 0.43, NS	*F*(9,135) = 0.92, NS
Omissions	*F*(3,135) = 59.97, *p* < 0.001	*F*(3,45) = 0.69, NS	*F*(9,135) = 1.21, NS
Premature	*F*(3,135) = 131.35, *p* < 0.001	*F*(3,45) = 4.25, *p* < 0.01	*F*(9,135) = 4.05, *p* < 0.001
Perseverant	*F*(3,135) = 0.44, NS	*F*(3,45) = 0.41, NS	*F*(9,135) = 1.55, NS
Latency reward	*F*(3,135) = 8.86, *p* < 0.001	*F*(3,45) = 0.31, NS	*F*(9,135) = 0.66, NS
Latency correct	*F*(3,135) = 9.35, *p* < 0.001	*F*(3,45) = 0.44, NS	*F*(9,135) = 1.11, NS
Latency incorrect	*F*(3,135) = 0.25, NS	*F*(3,45) = 0.15, NS	*F*(9,135) = 1.43, NS

**FIGURE 5:**
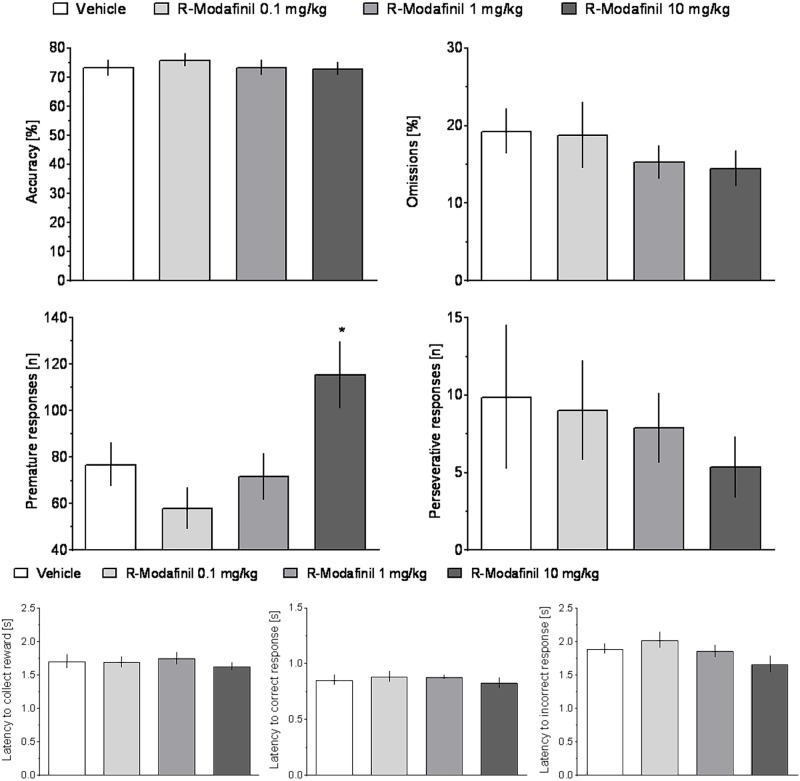
The effects of R-Modafinil on rat performance in the 5-CSRTT. R-Modafinil (0, 0.1, 1, or 10 mg/kg) was administered to rats IP 30 min before the task. *N* = 12–13 rats per group. Sprague-Dawley rats were exposed to vITI durations (2.5, 5, 7.5, and 10 s), equal numbers (i.e., 25) of each of the four ITIs were randomly presented during the 100 trial session over a 60-min period. The results represent the means ± SEM for total session without distinction on the individual ITIs.

**FIGURE 6:**
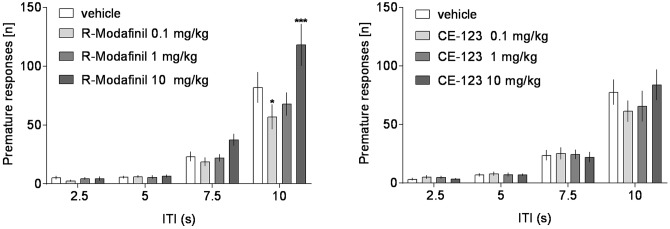
The effects of CE-123 and R-Modafinil on premature responding across vITIs. CE-123 (left) or R-Modafinil (0, 0.1, 1, or 10 mg/kg) was administered to rats IP 30 min before the task. *N* = 12–13 rats per group. Sprague-Dawley rats were exposed to vITI durations (2.5, 5, 7.5, and 10 s), equal numbers (i.e., 25) of each of the four ITIs were randomly presented during the 100 trial session over a 60-min period. The results represent the means ± SEM for the number of premature responses.

Analysis of premature responding revealed a significant effect of treatment and a significant treatment × ITI interaction in rats treated with R-Modafinil (**Figure 5** and **Table 1**). *Post hoc* analysis demonstrated that R-Modafinil administered at 10 mg/kg increased premature responding during ITI = 10 s, whereas decreases were noted when the drug was administered at a lower dose of 0.1 mg/kg. No such differences could be observed in CE-123-treated rats (**Figure 6**). ANOVA did not reveal significant effects of treatment or treatment × ITI interaction on any other measures (**Table 1**).

## Discussion

The S-form of 5-((benzhydrylsulfinyl)methyl)thiazole was obtained at high purity as shown by mass spectrometry, NMR, and HPLC. Intraperitoneal administration of this compound showed reduced ED shifts in the ASST in CE-123-treated rats at the two higher doses used (0.3 mg and 1 mg/kg bodyweight) as compared to controls, indicating increased cognitive flexibility. In the 5-CSRTT no treatment effect at any measure and any dosage could be determined in CE-123-treated rats, whereas in R-Modafinil-treated rats at the highest dose a significant treatment effect for premature responses at the ITI of 10 s was revealed. Thus, these rats showed increased impulsivity, an unwanted side effect, in contrast to CE-123-treated rats. The data confirm the view that cognitive flexibility is regulated in a linear dose-dependent pattern (Fallon et al., 2015), and suggest that that optimal performance is at higher dopamine levels. Besides the role of D1R there is also evidence that the D2R regulates ED performance (Mehta et al., 1999; Floresco, 2013; Gauthier et al., 2014; Nikiforuk et al., 2016). Most likely set shifting is modulated by interaction processes of several dopamine receptor subtypes (Floresco et al., 2006) and act synergistically with NMDAR in the processing of executive function in the ASST (Talpos and Shoaib, 2015; Desai et al., 2017). The involvement of DAT in ED set shifting is unclear but DAT is involved in several parameters in set shifting tasks. Cybulska-Klosowicz et al. (2017) found ID and reversal learning impairment in DAT heterozygous mutant mice, whereas Schulz et al. (2012) did not find any relation with allelic DAT variation and cognitive flexibility.

Modafinil at a single dose of 200 mg does not affect attentional set shifting in humans with first episode psychosis (Scoriels et al., 2012) and at the same dose it does not affect cognitive functions (Randall et al., 2003). However, in patients with chronic schizophrenia a single oral dose of 200 mg of Modafinil improved attentional set shifting and cognition (Turner et al., 2004b). Within this relatively high range of dosages it can even counteract executive functions, healthy humans acutely treated with 200 mg Modafinil made more total errors than placebo controls and subjects treated with 100 mg Modafinil in an intra-ED set shifting task (Randall et al., 2004). This is in contrast to the present results where even lower doses improved cognitive flexibility. The present results suggest that these tests are suitable in detecting low dosage effects on cognitive execution. Deficits in the ED shift stages induced by chronic infusion of phencyclidine in rats could be reversed by treatment with Modafinil at a dose of 64 mg/kg bodyweight, whereas the effect on control animals was not determined (Pedersen et al., 2009).

Generally, there is a very limited body of literature involving set shifting tasks in testing cognitive enhancing drugs in cognitively unimpaired rodents.

Impulsive responding in humans has been found to be reduced by Modafinil at a dose of 400 mg (Turner et al., 2003) in a stop-signal (stop) task. Attentional visual flexibility (shape and color) during learning was improved by an acute dose of 100 mg in humans (Pringle et al., 2013), suggesting that pharmacological effects upon set shifting may also depend on the stimuli modalities that are involved. The lack of treatment effects upon accuracy in the 5-CSRTT for both drugs may be related to the finding that attention in this task is mainly regulated by the cholinergic system (Muir et al., 1992; Jones and Higgins, 1995; Passetti et al., 2000). Waters et al. (2005) did not find effects on accuracy or omission in a 5-CSRTT in rats acutely treated with Modafinil at 32–128 mg/kg bodyweight, but similar to our results impulsive responding was increased under increased attentional load, or sustained attention during increased length of the ITI as in our case. Also, scopolamine-induced impairment of 5-CSRTT performance could not be reversed by Modafinil treatment. Dopaminergic effects are mainly related to speed and probability of responding (Baunez and Robbins, 1999; Milstein et al., 2003), whereas improvement of accuracy could be observed mainly in rodents with low baseline performance (Granon et al., 2000). Modafinil has sympathomimetic effects and can directly influence the locus coeruleus activity, the main source of noradrenaline in the brain, via activation of hypocretin (Ivanov and Aston-Jones, 2000; Chudasama and Robbins, 2004). Noradrenaline is known to enhance attentional performance in the 5-CSRTT (Robbins, 2002), but the effects depend on task contingencies (Dalley et al., 2001).

## Conclusion

The enantiomeric S-form of CE-123 enhanced cognitive flexibility at very low dosages without producing impulsive responding even at higher dosages and therefore may be suitable for further testing of its cognition enhancing properties in different behavioral paradigms.

## Author Contributions

GL proposed and designed the study. PP and AN designed the study and wrote the protocol. JG carried out the experiments. VK has mainly written the manuscript. PK and VD carried out the synthesis. JW carried out analytical procedures. TL contributed to the synthesis of the compounds. MI carried out and analyzed the reuptale assays. HH contributed to interpretation and writing the manuscript. All authors contributed to and have approved the final manuscript.

## Conflict of Interest Statement

The authors declare that the research was conducted in the absence of any commercial or financial relationships that could be construed as a potential conflict of interest.
