# Supplementary material for: A Novel Dopamine Transporter Inhibitor CE-123 Improves Cognitive Flexibility and Maintains Impulsivity in Healthy Male Rats
Source: Front Behav Neurosci. 2017 Nov 27;11:222. doi: 10.3389/fnbeh.2017.00222 (PMC5711856; doi:10.3389/fnbeh.2017.00222)
Supplement: Supplementary file 1 [file Table_1.PDF]

PK parameters of R-Modafinil after ip dosing of 10 mg/kg R-Modafinil

R-ModafinilIP							
	Unit	#279	#278	#284	Mean	SD	CV%
Dose	mg/kg	10	10	10	10	0	0
Cmax	ng/mL	6818.52	7886.25	1942.16	5548.98	3168.89	57.11
Tmax	h	0.08	0.08	0.08	0.08	0.00	0.00
Cz(Obs)	ng/mL	42.0	17.8	16.4	25.4	14.4	56.6
Tz	h	2.0	2.0	4.0	2.7	1.2	43.3
t1/2	h	0.3	0.3	0.7	0.4	0.2	57.9
AUC(0-tz)	ng/mL*h	1994.3	2213.1	1158.9	1788.8	556.4	31.1
AUC(tz-inf)	ng/mL*h	18.2	6.5	16.4	13.7	6.3	46.0
AUC(0-inf)	ng/mL*h	2012.5	2219.6	1175.3	1802.5	553.0	30.7
%AUCextra		0.90	0.29	1.39			
Vz/f	mL/kg	2156.1	1643.8	8475.8	4091.9	3805.2	93.0
CL/f	mL/h/kg	4968.9	4505.3	8508.8	5994.3	2189.9	36.5
R^2		0.975	0.999	0.993			

PK parameters of CE-123 after ip dosing of 10 mg/kg CE-123 enantiomer S

CE-123 enantiomer IIP						
	Unit	#281	#285	Mean	SD	CV%
Dose	mg/kg	10	10			
Cmax	ng/mL	9586.74	1698.17			
Tmax	h	0.08	0.08			
Cz(Obs)	ng/mL	277.0	279.3			
Tz	h	7.0	7.0			
t1/2	h	1.5	4.1			
AUC(0-tz)	ng/mL*h	18710.3	5812.4			
AUC(tz-inf)	ng/mL*h	591.0	1634.9			
AUC(0-inf)	ng/mL*h	19301.3	7447.3			
%AUCextra		3.06	21.95			
Vz/f	mL/kg	1105.3	7859.3			
CL/f	mL/h/kg	518.1	1342.8			
R^2		0.986	0.750			

#285 was considered as failed intraperitoneal administration. Means were not calculated.

CSF/plasma concentration ratio of R-Modafinil

Time (h)	Rat ID	CSF conc. [ng/ml]	Mean [ng/ml]	SD [ng/ml]	CV%	CSF/Plasma ratio	Mean	SD	CV%
0.25	#286<br>#287	663.8<br>385.2	524.5	197.0	37.6	0.22<br>0.25	0.23	0.02	8.40
1	#288<br>#289	136.5<br>90.6	113.5	32.4	28.6	0.52<br>0.66	0.59	0.09	15.92
7	#279<br>#278<br>#284	3.45<br>0.00*<br>0.00*	1.2	2.0	173.2	-<br>-<br>-	-	-	-

*not found, set to zero

Brain/plasma concentration ratio of R-Modafinil

Time (h)	Rat ID	Brain conc. [ng/g]	Mean [ng/g]	SD [ng/g]	CV%	Brain/Plasma ratio	Mean	SD	CV%
0.25	#286<br>#287	1272.0<br>905.6	1088.8	259.1	23.8	0.42<br>0.58	0.50	0.11	22.68
1	#288<br>#289	114.2<br>121.2	117.7	4.9	4.2	0.44<br>0.88	0.66	0.31	47.28
7	#279<br>#278<br>#284	0.00*<br>0.00*<br>0.00*	0.0	0.0	-	-<br>-<br>-	-	-	-

*not found, set to zero

CSF/plasma concentration ratio of CE-123 following ip dosing of CE-123 enantiomer S

Time (h)	Rat ID	CSF conc. [ng/ml]	Mean [ng/ml]	SD [ng/ml]	CV%	CSF/Plasma ratio	Mean	SD	CV%
0.25	#294	570.0	513.2	80.3	15.7	0.10	0.11	0.00	1.04
	#295	456.4				0.11			
1	#296	148.0	150.6	3.7	2.5	0.14	0.12	0.03	22.62
	#297	153.3				0.10			
7	#281	23.0	27.8	6.9	24.7	0.08	0.10	0.02	24.18
	#285	32.7				0.12			

Brain/plasma concentration ratio of CE-123 following ip dosing of CE-123 enantiomer S

Time (h)	Rat ID	Brain conc. [ng/g]	Mean [ng/g]	SD [ng/g]	CV%	Brain/Plasma ratio	Mean	SD	CV%
0.25	#294	3512.7	3320.2	272.3	8.2	0.64	0.69	0.06	8.53
	#295	3127.7				0.73			
1	#296	706.0	869.1	230.8	26.6	0.68	0.68	0.01	1.57
	#297	1032.3				0.69			
7	#281	197.0	212.8	21.1	9.9	0.71	0.76	0.07	9.31

Supplementary table 1. Ratio of brain/plasma concentrations of R-Modafinil and CE-123 racemate after i.p. administration of 10 mg/kg bodyweight.

Concentration of the test items was measured in order to assess the brain penetration of the test items. CSF concentration is considered as a measure of the free (pharmacologically active) drug concentration in brain, whereas brain tissue concentration is considered as a measure of the total concentration of test item in brain. The data are reported in the following tables.

The ratios were more or less identical for the 3 test items. However, in accordance with the longer plasma half life of CE-123, CSF and brain exposure as such were higher for CE-123, compared to Modafinil.

Abbreveations:

Cmax (ng/mL) maximal observed concentration
Tmax (h) time of maximal observed concentration
Cz (ng/ml) last analytically quantifiable concentration
Tz (h) time of the last sample which has an analytically quantifiable concentration
t1/2z (h) half life of the terminal slope of a concentration-time curve
AUC$_{0\text{-}tz}$ (ng*h/ml) area under the concentration-time curve up to the time t_z of the last sample
AUC$_{tz\text{-}inf}$ (ng*h/ml) area under the concentration-time curve extrapolated from t_z to infinity
AUC$_{0\text{-}\infty}$ (ng*h/ml) area under the concentration-time curve extrapolated to infinity
Vz/f (ml/kg) volume of distribution not normalized by bioavailability
CL/f (ml/(h*kg)) total body clearance not normalized by bioavailability
R^2 coefficient of regression

The analyses were done by Pharmacelsus, Contract Research Organisation, Saarbrücken, Germany.
