# Supplementary material for: A Novel Dopamine Transporter Inhibitor CE-123 Improves Cognitive Flexibility and Maintains Impulsivity in Healthy Male Rats
Source: Front Behav Neurosci. 2017 Nov 27;11:222. doi: 10.3389/fnbeh.2017.00222 (PMC5711856; doi:10.3389/fnbeh.2017.00222)
Supplement: Supplementary file 2 [file Data_Sheet_1.PDF]

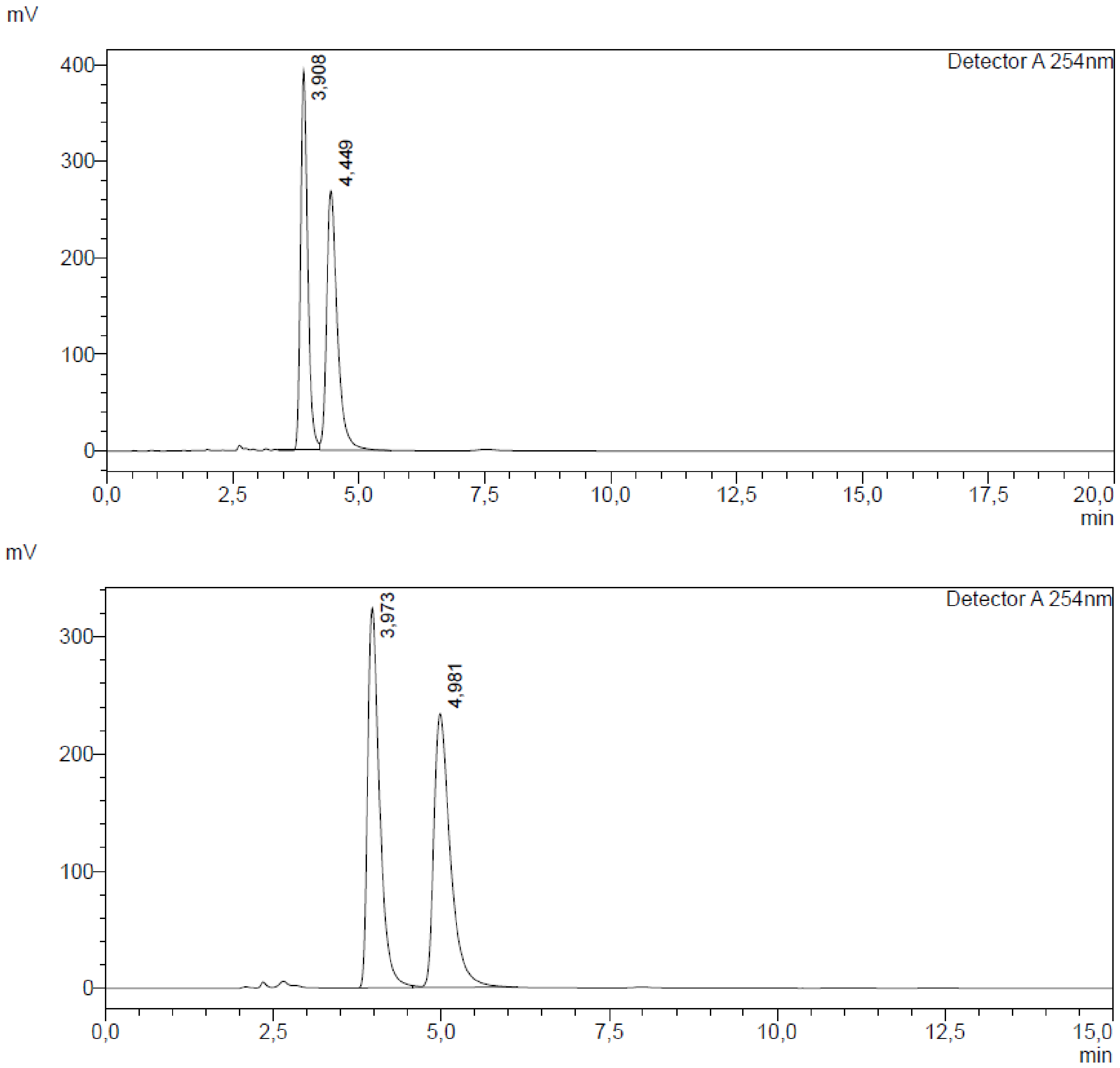

Supplementary Figure 1. Method development for chiral separations of CE-123 enantiomers.

Screening was performed on ChiralpackIA, Chiralpack IC and Chiralpack ADH columns. The best conditions were obtained for Chiralpack IA column with 100% acetonitrile (chromatogram above) and 100% ethanol (chromatogram below).

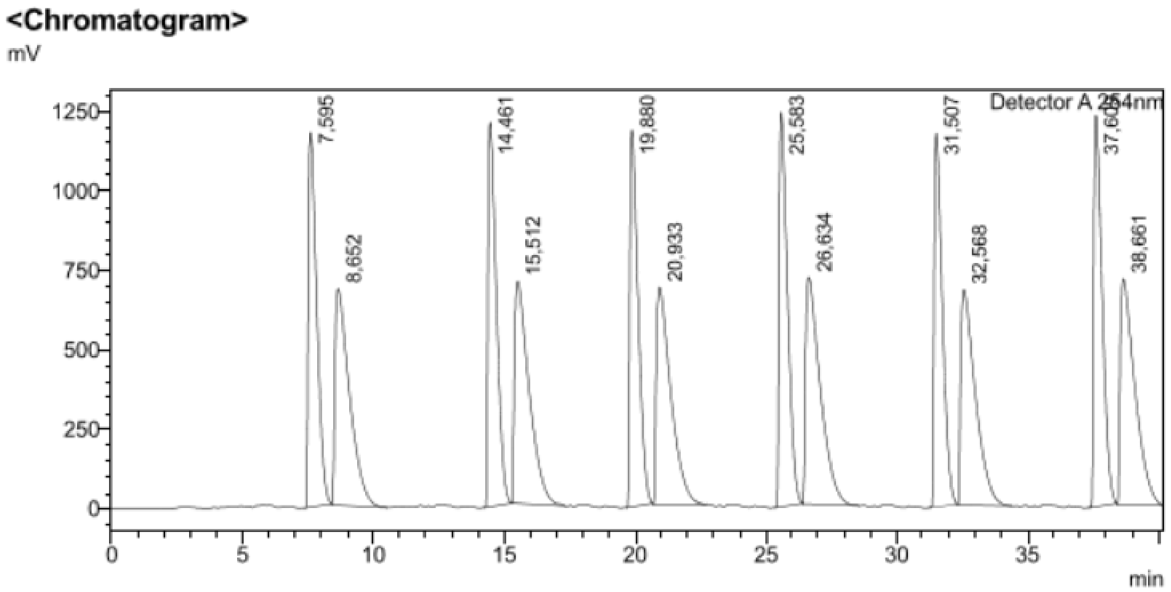

Supplementary Figure 2. Separation of CE-123 enantiomers using semi-preparative column.

Stacked-injections were performed on semi-preparative ChiralpackIA column using 100% ethanol as a mobile phase. Fractions containing same enantiomers were pooled together, concentrated and dried under reduced pressure to obtain required amount of the desired enantiomers.

<Chromatogram>

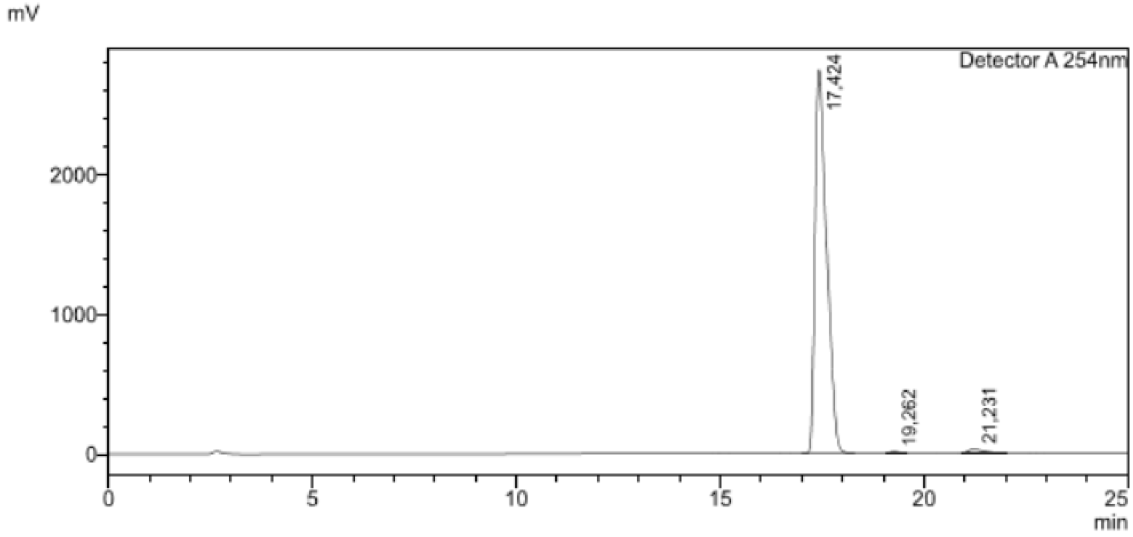

<Peak Table>

Detector A 254nm

Peak#	Ret. Time	Area	Conc.	Area%
1	17,424	54924910	98,344	98,344
2	19,262	156436	0,280	0,280
3	21,231	768701	1,376	1,376
Total		55850048		100,000

Supplementary Figure 3. Purity of the racemic CE-123 preparation.

Purity of the racemic CE-123 was determined by using a HPLC-based method with Acclaim 120 C18, 2.1 x 150 mm, 3 µm HPLC column (Thermo Fisher Scientific). Determined retention time 17.4 min, purity 98.3%.

Acquisition Parameter

Source Type	ESI	Ion Polarity	Positive	Set Nebulizer	0.4 Bar
Focus	Active	Set Capillary	1300 V	Set Dry Heater	200 °C
Scan Begin	50 m/z	Set End Plate Offset	-500 V	Set Dry Gas	4.0 l/min
Scan End	1550 m/z	Set Charging Voltage	2000 V	Set Divert Valve	Waste
		Set Corona	0 nA	Set APCI Heater	0 °C

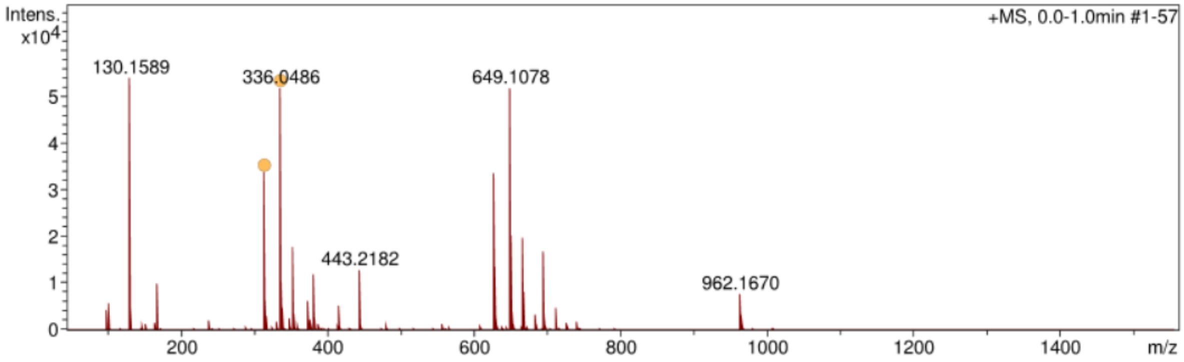

Meas. m/z	#	Ion Formula	m/z	err [ppm]	mSigma	# mSigma	Score	rdb	e⁻ Conf	N-Rule
314.0667	1	C17H16NOS2	314.0668	0.3	12.9	1	100.00	10.5	even	ok
	2	C17H8N5O2	314.0673	1.8	38.2	2	33.83	16.5	even	ok
336.0486	1	C17H15NNaOS2	336.0487	0.4	9.9	1	100.00	10.5	even	ok

Supplementary Figure 4. HRESIMS spectra of the racemic CE-123 preparation.

Experimentaly determined molecular mass of the CE-123 from the precursor ion is *m/z*314.0667 $[M+H]^+$ (calculated for $C_{17}H_{16}NOS_2^+$, 314.0668, $\Delta = 0.3$ ppm).

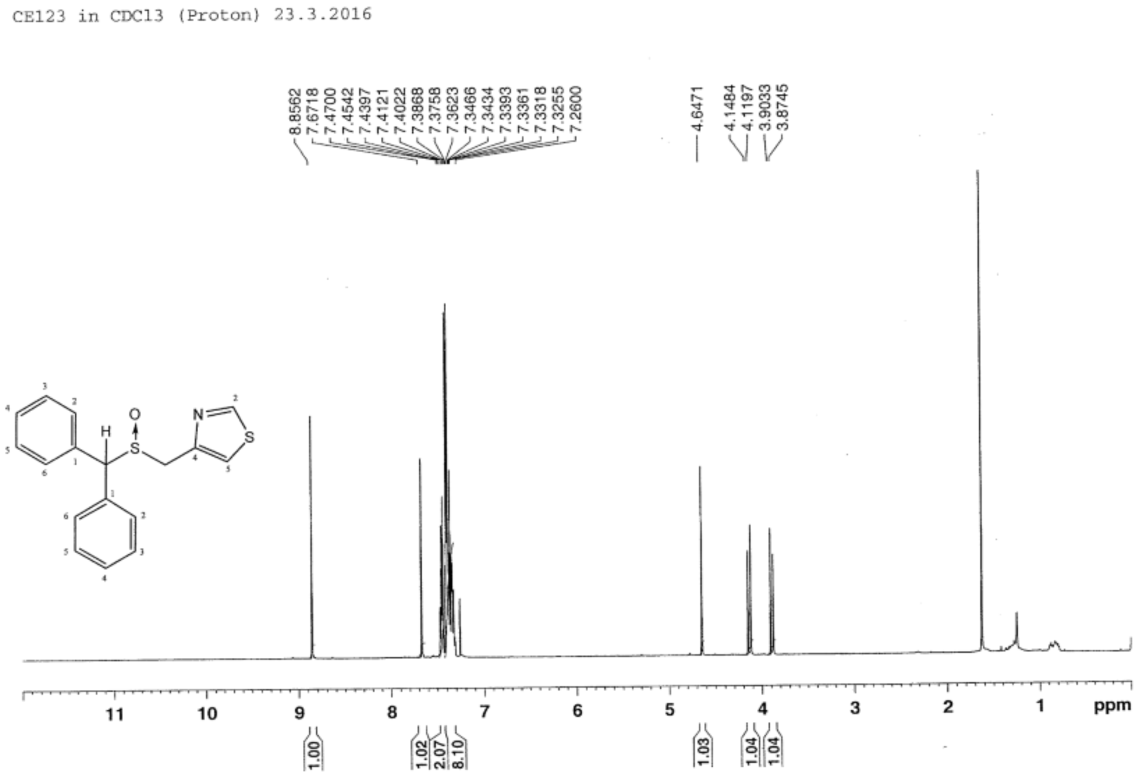

Supplementary Figure 5. Proton NMR spectra of the racemic CE-123 preparation.

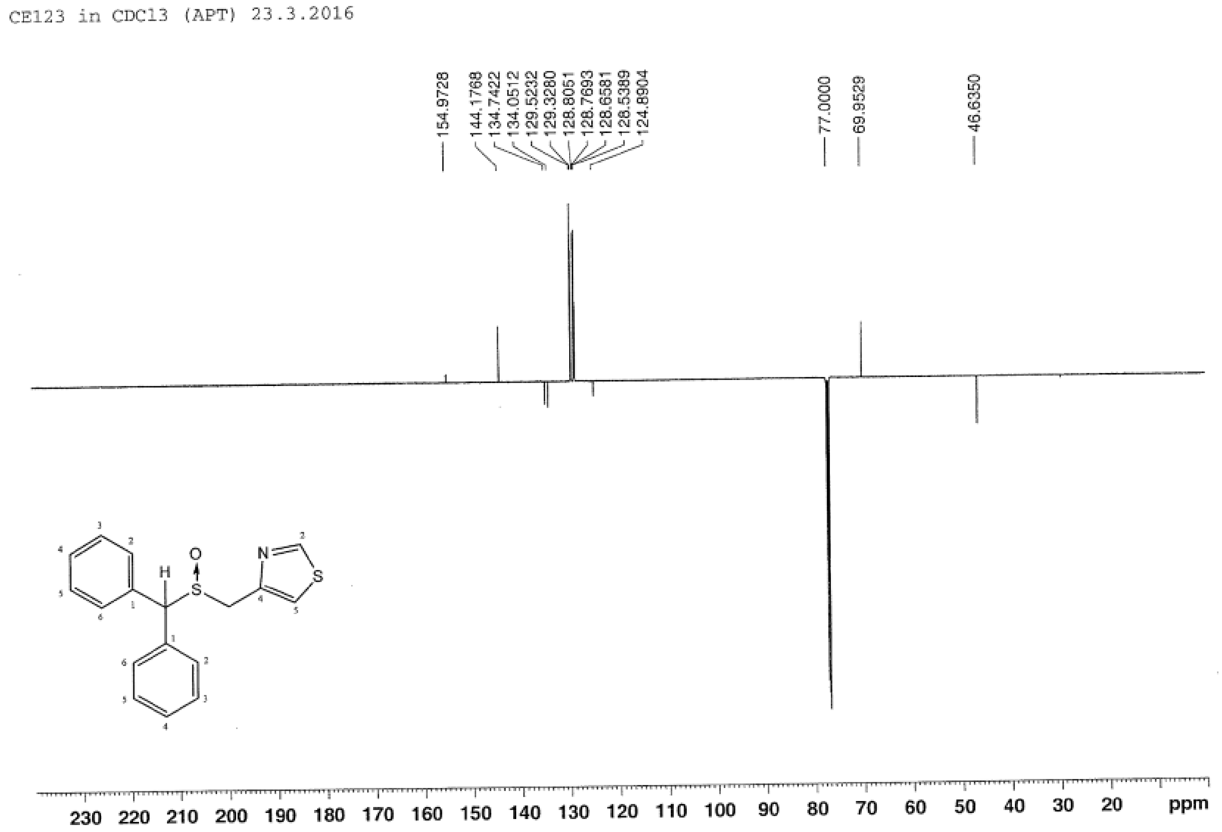

Supplementary Figure 6. Carbon NMR spectra of the racemic CE-123 preparation.

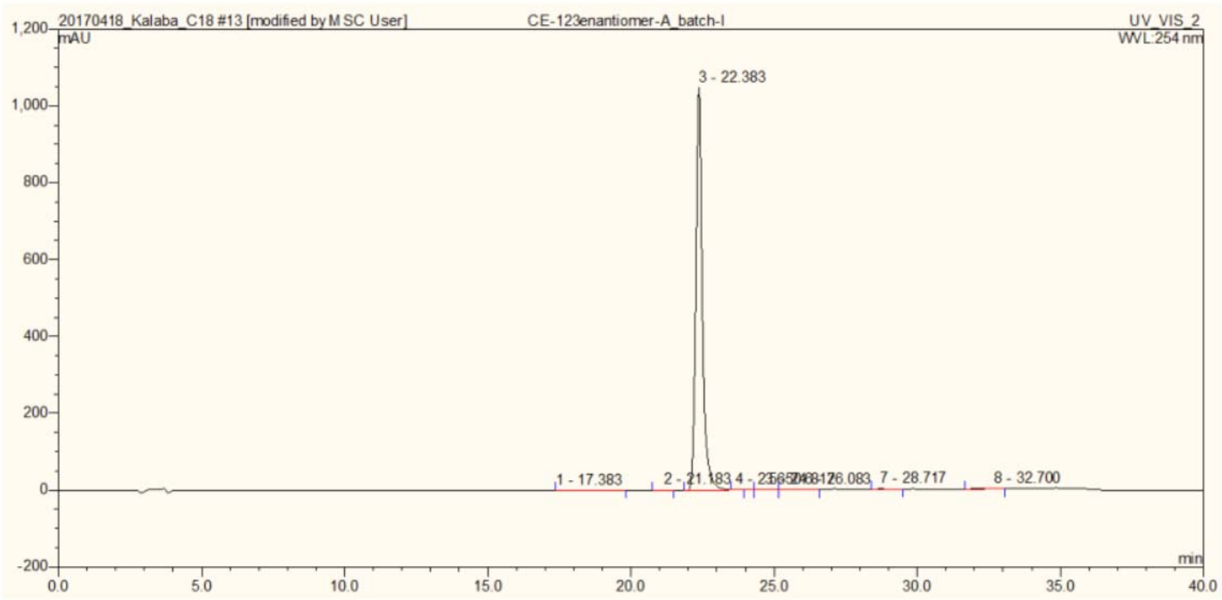

Supplementary Figure 7. Purity of the less retained enantiomer S.

Purity of the CE-123less retained enantiomer S was determined by using a HPLC-based method with Acclaim 120 C18, 2.1 x 150 mm, 3 μm HPLC column (Thermo Fisher Scientific). Determined purity 99.4%.

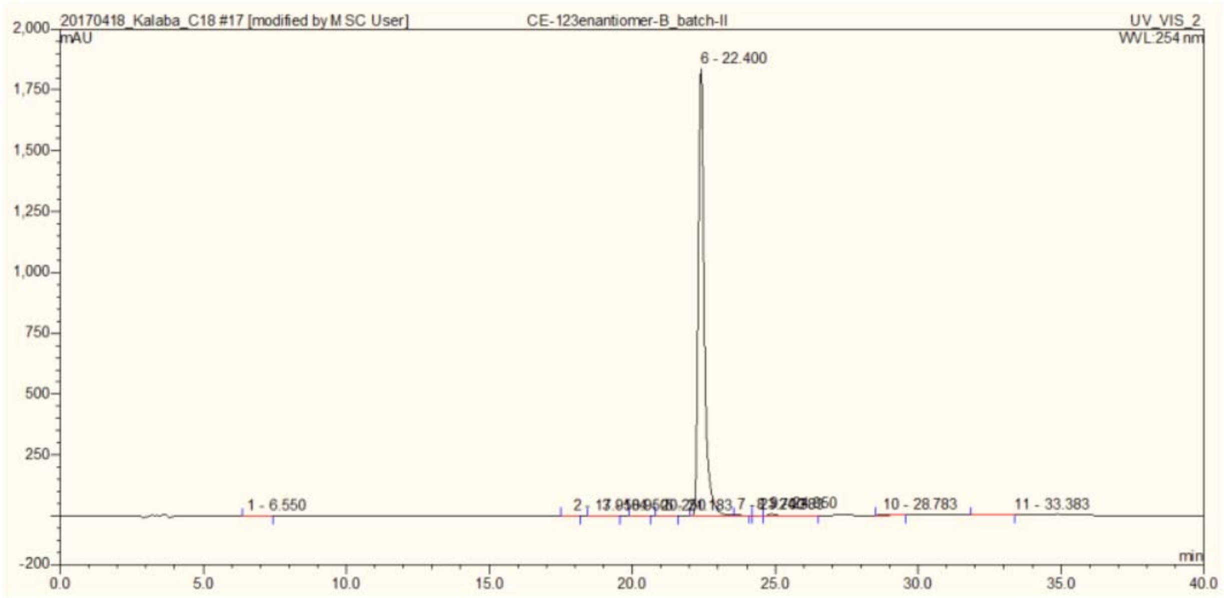

Supplementary Figure. 8. Purity of the more retained enantiomer R.

Purity of the CE-123more retained enantiomer R was determined by using a HPLC-based method with Acclaim 120 C18, 2.1 x 150 mm, 3 μm HPLC column (Thermo Fisher Scientific). Determined purity 99.2%.

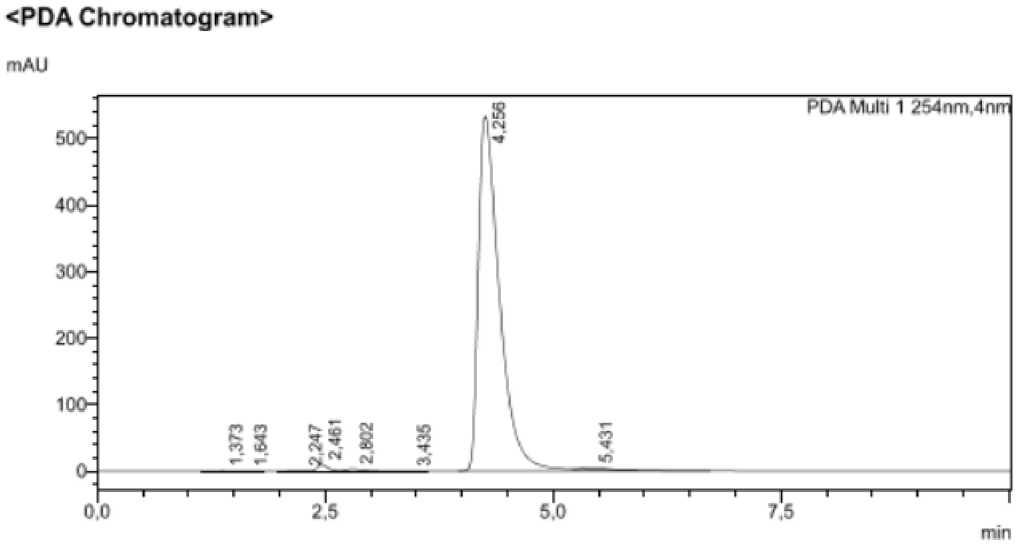

Peak Table

PDA Ch1 254nm

Peak#	Ret. Time	Area	Area%
1	1,373	3041	0,034
2	1,643	1605	0,018
3	2,247	6189	0,070
4	2,461	78456	0,882
5	2,802	40391	0,454
6	3,435	1585	0,018
7	4,256	8719185	97,979
8	5,431	48576	0,546
Total		8899028	100,000

Supplementary Figure 9.Enantio-purity of the CE-123 enantiomer S.

Enantio-purity of the enantiomer S was determined to be 98% using a HPLC method with analytical ChiralpackIA column and 100% ethanol.

<PDA Chromatogram>

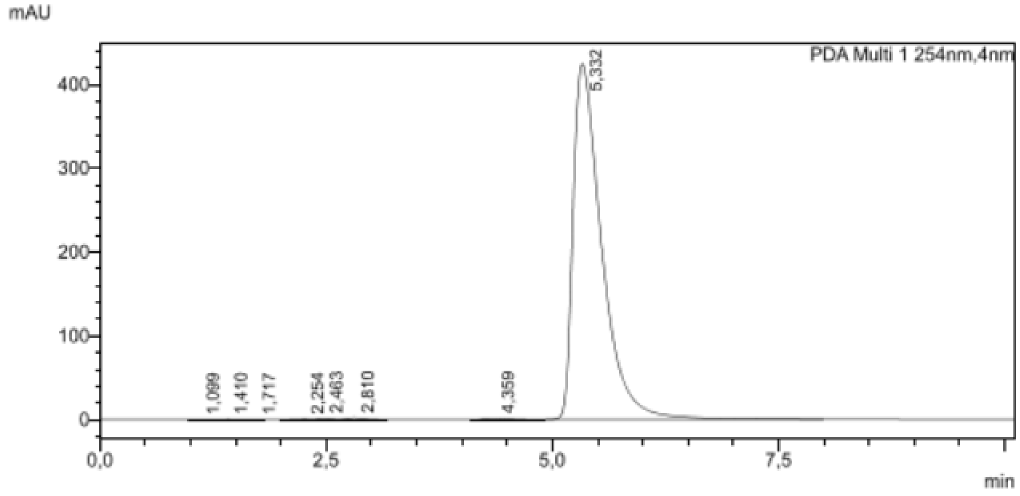

Peak Table

PDA Ch1 254nm

Peak#	Ret. Time	Area	Area%
1	1,099	1272	0,014
2	1,410	2806	0,030
3	1,717	1527	0,016
4	2,254	5937	0,063
5	2,463	15891	0,169
6	2,810	23190	0,247
7	4,359	29620	0,315
8	5,332	9322395	99,147
Total		9402638	100,000

Supplementary Figure 10. Enantio-purity of the CE-123 enantiomer R.

Enantio-purity of the enantiomer R was determined to be 99% using a HPLC method with analytical ChiralpackIA column and 100% ethanol.

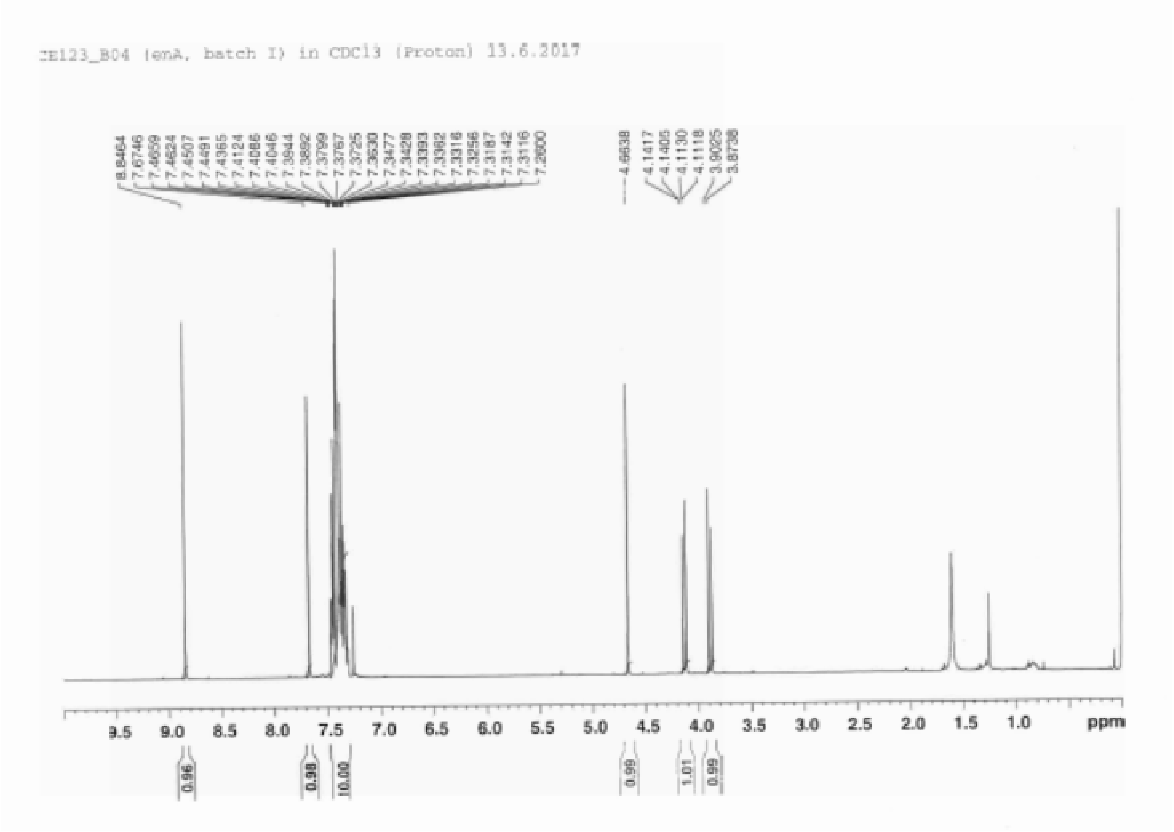

Supplementary Figure 11.Proton NMR spectra of the less retained enantiomer S.

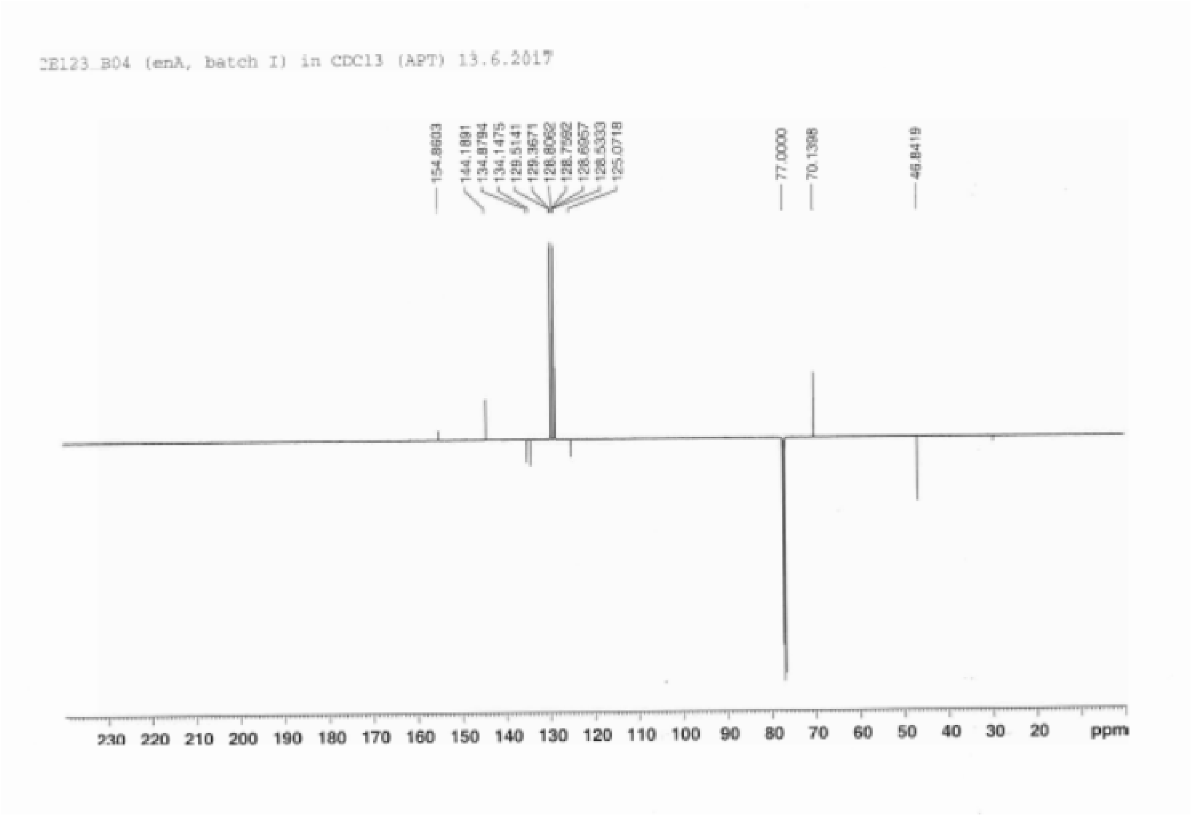

Supplementary Figure 12. Carbon NMR spectra of the less retained enantiomer S.

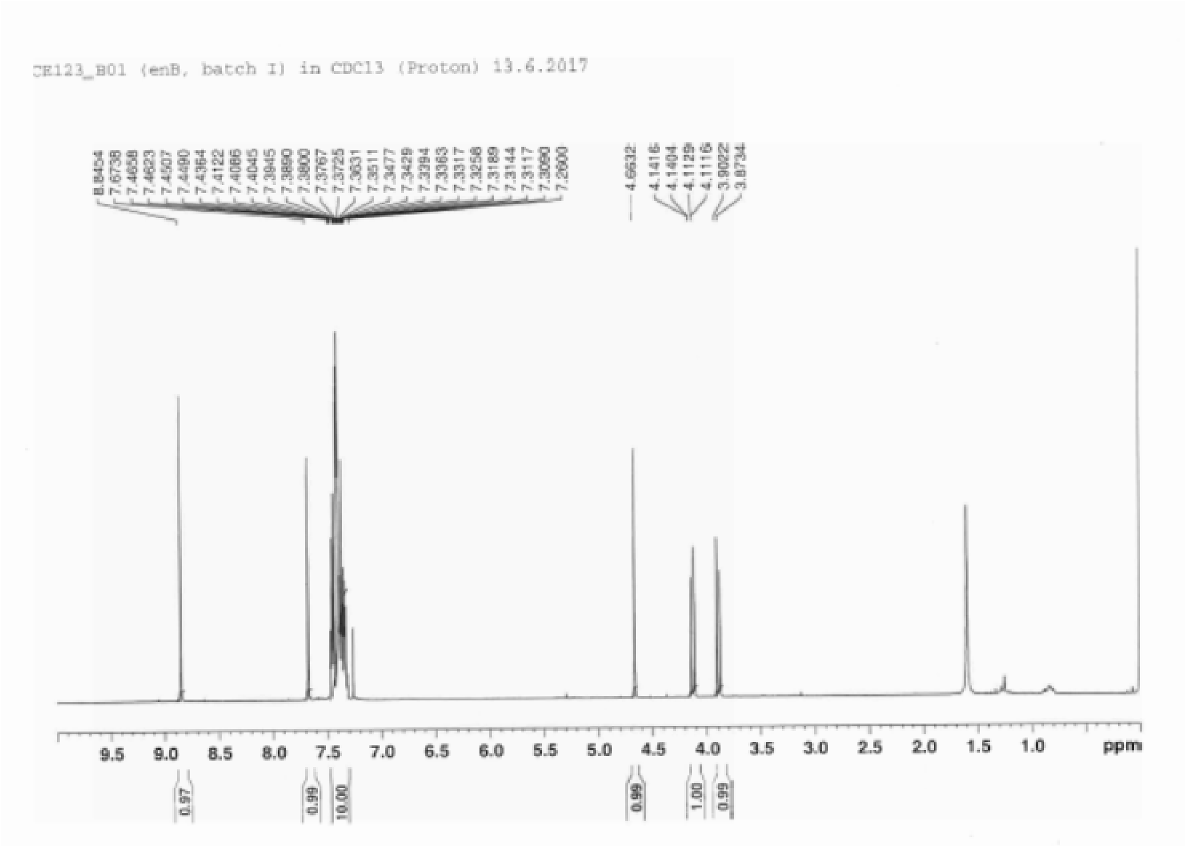

Supplementary Figure 13.Proton NMR spectra of the more retained enantiomer R.

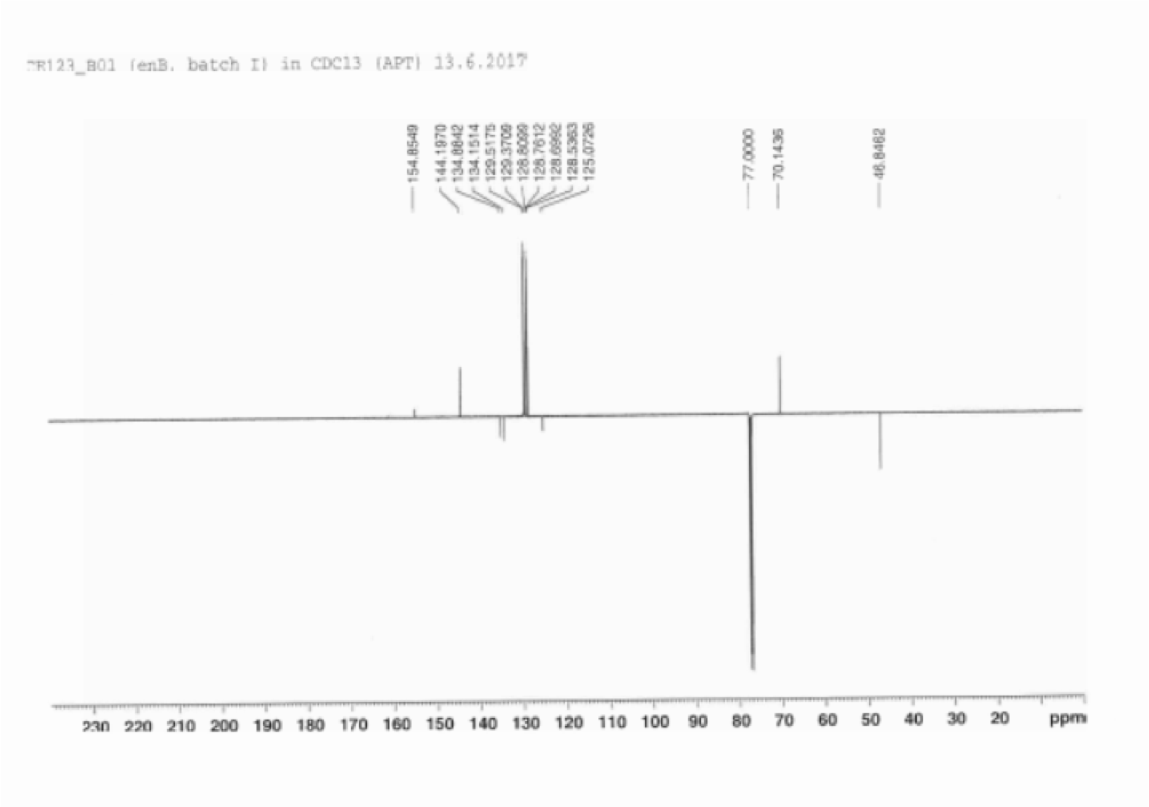

Supplementary Figure 14. Carbon NMR spectra of the more retained enantiomer R.

Acquisition Parameter

Source Type	ESI	Ion Polarity	Positive	Set Nebulizer	2.0 Bar
Focus	Not active	Set Capillary	4500 V	Set Dry Heater	200 °C
Scan Begin	50 m/z	Set End Plate Offset	-500 V	Set Dry Gas	4.0 l/min
Scan End	1600 m/z	Set Collision Cell RF	500.0 Vpp	Set Divert Valve	Source

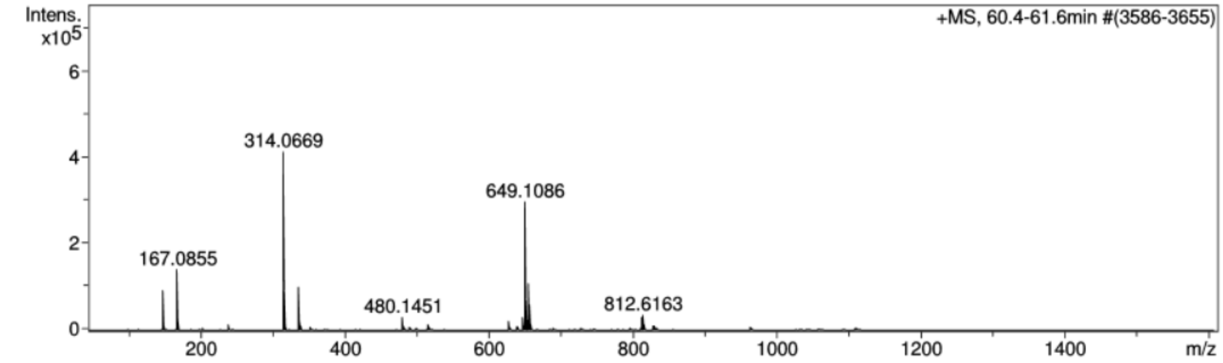

Meas. m/z	#	Formula	Score	m/z	err [mDa]	err [ppm]	mSigma	rdb	e⁻ Conf	N-Rule
314.0669	1	C 17 H 16 N O S 2	100.00	314.0668	-0.2	-0.5	10.4	10.5	even	ok
	2	C 17 H 8 N 5 O 2	80.88	314.0673	0.3	1.0	18.1	16.5	even	ok
	3	C 16 H 12 N O 6	23.63	314.0659	-1.0	-3.3	51.4	11.5	even	ok
336.0488	1	C 17 H 15 N Na O S 2	100.00	336.0487	-0.0	-0.1	8.3	10.5	even	ok
	2	C 17 H 7 N 5 Na O 2	74.93	336.0492	0.4	1.3	14.0	16.5	even	ok

Supplementary Figure 15. HRESIMS spectra of the less retained enantiomer S.

Experimentaly determined molecular mass of the CE-123 enantiomer S from the precursor ion is *m/z*314.0669[M+H]$^+$ (calculated for $C_{17}H_{16}NOS_2^+$, 314.0668, Δ = -0.5 ppm).

Acquisition Parameter

Source Type	ESI	Ion Polarity	Positive	Set Nebulizer	2.0 Bar
Focus	Not active	Set Capillary	4500 V	Set Dry Heater	200 °C
Scan Begin	50 m/z	Set End Plate Offset	-500 V	Set Dry Gas	4.0 l/min
Scan End	1600 m/z	Set Collision Cell RF	500.0 Vpp	Set Divert Valve	Source

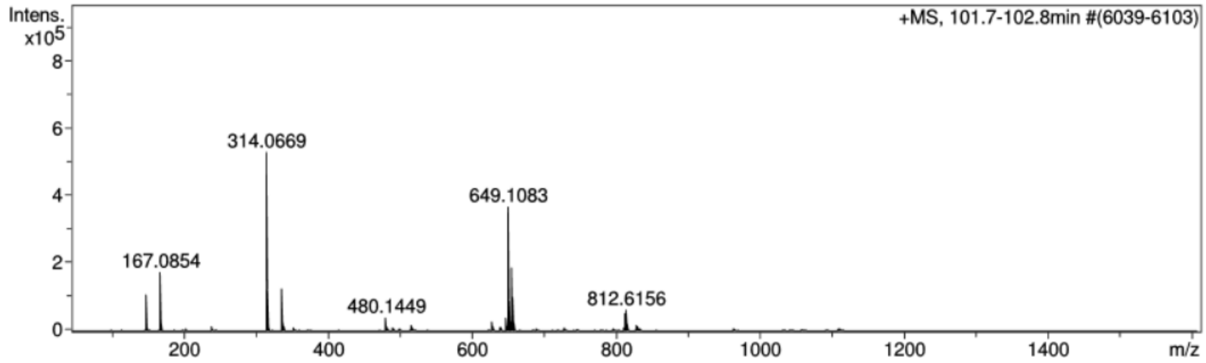

Meas. m/z	#	Formula	Score	m/z	err [mDa]	err [ppm]	mSigma	rdb	e⁻ Conf	N-Rule
314.0669	1	C 17 H 16 N O S 2	100.00	314.0668	-0.1	-0.3	13.7	10.5	even	ok
	2	C 17 H 8 N 5 O 2	75.38	314.0673	0.4	1.2	21.2	16.5	even	ok
	3	C 16 H 12 N O 6	22.53	314.0659	-1.0	-3.0	55.2	11.5	even	ok
336.0486	1	C 17 H 15 N Na O S 2	100.00	336.0487	0.1	0.2	8.0	10.5	even	ok
	2	C 17 H 7 N 5 Na O 2	72.44	336.0492	0.5	1.6	13.9	16.5	even	ok

Supplementary Figure 16. HRESIMS spectra of the more retained enantiomer R.

Experimentaly determined molecular mass of the CE-123 enantiomer R from the precursor ion is *m/z*314.0669$[M+H]^+$ (calculated for $C_{17}H_{16}NOS_2^+$, 314.0668, Δ = -0.1 ppm).

A

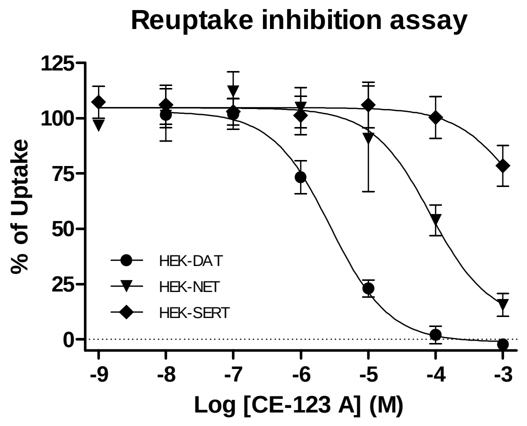

	HEK-DAT	HEK-NET	HEK-SERT
One site competition			
Best-fit values			
Bottom	-1.165	8.623	42.66
Top	103.0	104.7	104.7
LogEC50	-5.559	-4.077	-2.866
EC50	2.762e-006	8.371e-005	0.001362

B

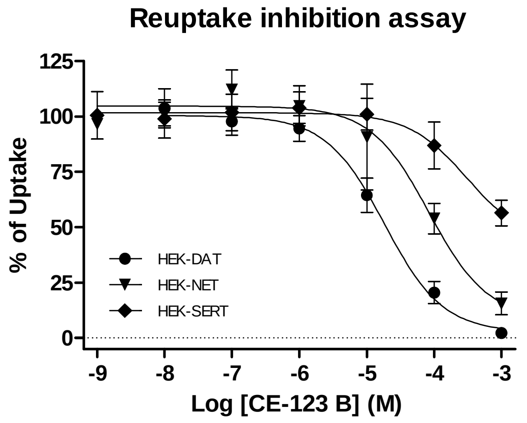

	HEK-DAT	HEK-NET	HEK-SERT
One site competition			
Best-fit values			
Bottom	2.551	8.623	42.49
Top	100.4	104.7	101.7
LogEC50	-4.739	-4.077	-3.512
EC50	1.823e-005	8.371e-005	0.0003078

Supplementary Figure 17. Reuptake inhibiton assay for both enantiomers of CE-123.

Reuptake inhibition assay. Inhibition of [^{3}H]DA, [^{3}H]MPP+ and [^{3}H]5-HT reuptake by increasing concentrations of CE-123 enantiomer S (A) and enatiomer R (B) in HEK293 cells stably expressing human isoforms of DAT, NET and SERT. Experiments were performed as described in Materials and Methods. Nonspecific uptake was defined in the presence of10 µMmazindolfor HEK-DAT and HEK-NET and 10 µM paroxetine for HEK-SERT. Data are means ± S.D. of 3-4 experiments performed in duplicates or triplicates.

Data analysis

The IC_{50} values were determined by nonlinear regression of experimental data for each experiment using the computer program GraphPad Prism 5. Enantiomer S (En S), Enantiomer R (En R).

Values with SD

	CE-123					
	DAT		NET		SERT	
	En S	En R	En S	EnR	En S	En R
	2,301	17,24	150,8	95,36	Not specific	Not specific
	2,521	15,95	134,7	79,84	Not specific	Not specific
	3,04	18,85	127,6	77,06	Not specific	Not specific
	2,841	22,32				
SD	0,32882151	2,75515275	11,8873883	9,86144682	Not specific	Not specific
